# A New Real-Life Test for Reciprocity in Autistic Adults: The Interactive Drawing Test

**DOI:** 10.3389/fpsyt.2022.842902

**Published:** 2022-03-21

**Authors:** Tineke Backer van Ommeren, Marianne Vreugdenhil, Hans M. Koot, Annelies Spek, Anke M. Scheeren, Robert M Jertberg, Sander Begeer

**Affiliations:** ^1^Department of Clinical, Neuro and Developmental Psychology, Vrije Universiteit Amsterdam, and Amsterdam Public Health Research Institute, Amsterdam, Netherlands; ^2^Autism Expertise Center, Eemnes, Netherlands

**Keywords:** autistic adults, social interaction, reciprocity, real-life diagnostic assessment, engagement, online social cognition

## Abstract

Impaired social-emotional reciprocity is a defining feature of Autism Spectrum Disorder (ASD). Deficits in reciprocity can be difficult to assess, particularly in adults with average or above average intelligence. The recently developed Interactive Drawing Test (IDT) measures reciprocity in children and adolescents with and without ASD based on spontaneous non-verbal interactions during the joint creation of a drawing. In this study, we examined if the IDT is able to differentiate between 19 normally intelligent adults with ASD and 18 without ASD. The IDT total reciprocity score, including the number of meaningful contributions to objects initially drawn by the examiner, was lower in the autistic adults compared to those without ASD. By assessing both the quantity and quality of spontaneous reciprocal behavior, the IDT was able to identify subtle differences in reciprocal behavior of adults with and without ASD with average intelligence. Even though a larger sample is required to determine its psychometric properties, the IDT appears a promising tool to enrich the diagnostic process of ASD in adults.

Impaired social-emotional reciprocity is a defining feature of Autism Spectrum Disorder (ASD). The clinical field lacks sensitive tests for assessing impaired reciprocity. The recently developed Interactive Drawing Test (IDT) for reciprocity was tested in autistic and non-autistic adults. During the IDT, an examiner and participant make a joint drawing, taking turns, without specifying what they will draw. We aimed to investigated whether autistic adults showed less reciprocal behavior on the IDT compared to non-autistic participants. Autistic participants were less likely to jointly draw with the examiner, in particular when the examiner initiated a topic. The IDT revealed subtle but characteristic differences in reciprocal behavior related to ASD, suggesting it may be a promising diagnostic tool.

## Introduction

Impaired social-emotional reciprocity is one of the defining features of Autism Spectrum Disorder (ASD). The DSM-5 describes deficits in reciprocity as a necessary criterion for an ASD diagnosis “ranging from abnormal social approach and failure of normal back-and forth conversation, to reduced sharing of interests, emotions, or affect, and failure to initiate or respond to social interactions” (APA, 2013, p. 50) (1). Recent studies with a new instrument for measuring reciprocity in autistic individuals, the Interactive Drawing Test (IDT), have demonstrated that young autistic individuals (age range 6–18 years) show clear limitations in real-life reciprocal behavior compared to typically developing peers (2–4). While the IDT manual is published and used in Dutch mental health care clinics (5), the IDT has not yet been tested in autistic adults.

Validated instruments to measure social behavior of autistic or neurotypical adults are rare (6–9). Measuring social behavior is especially challenging in autistic adults with an average or higher IQ. These individuals are often able to camouflage or compensate for their social impairments by using their cognitive, analytical, and verbal abilities (10). This could make their impairments seem milder or even non-existent (11, 12). The assessment of real-life social interactions may be less sensitive to work around compensatory strategies (13). Consequently, it can take a long time before autistic adults with an average or high IQ are appropriately diagnosed (14). Furthermore, autistic adults are often either left undiagnosed or misdiagnosed, due to camouflaging or compensation behavior (10, 15) which is reason for great concern (6, 11, 16). A sensitive test for reciprocal behavior (a deficit in which is one of the defining features of ASD) could provide a welcome addition to the few existing diagnostic tests of ASD in adulthood.

Guidelines for diagnosing ASD in adults stress that more than one method of diagnostic assessment should be used, including parent/proxy report of early development and current functioning, supplemented by real-life tests of behavior (6, 17). However, retrospective parent reports of ASD symptoms in early childhood may be unreliable when administered to adults (18). Also, standardized questionnaires, such as the Social Responsiveness Scale and the Autism Quotient, require reflection on one's own abilities and behaviors. Reflecting and reporting on one's own reciprocal behavior may be difficult and subject to bias. Observations of real-life behavior may therefore provide an invaluable addition to the assessment of reciprocal behavior.

The Autism Diagnostic Observation Schedule [ADOS (19)], a widely used standardized diagnostic test for autism, provides multiple and diverse opportunities to show reciprocity during a semi-structured interaction with an examiner/clinician. Behaviors are carefully weighed by the examiner and translated into a binary score indicating presence/absence of adequate reciprocal behavior. However, though the ADOS has good predictive value for ASD, and includes a separate score for reciprocal information, the *quality* of reciprocal behavior and the *level of severity* of reciprocal impairments are not provided. Consequently, currently used diagnostic instruments may provide insufficient information about the nature and/or severity of impairments in reciprocity in ASD.

The interactive drawing test [IDT (2)] was developed to objectively measure the quality of reciprocity in a real-life interaction. During the IDT, the examiner and the client join in making a collaborative drawing. The examiner follows a semi-structured protocol, whereas the client receives only a general instruction: “We are going to draw together.” Different aspects of reciprocal drawing behavior are assessed and scored in the IDT. For instance, basic reciprocal behavior is operationalized by counting how often the client takes turns and imitates the behavior of the examiner. More advanced reciprocal behavior is operationalized by counting how often the client contributes meaningful elements to mutual drawing elements, for instance, adding wheels to a car. Importantly, we monitor the client's initiative during the interactions. We distinguish between client contributions in drawn objects initiated by the client or by the examiner. Finally, we monitor the flexibility with which the client responds to intrusive drawing actions of the examiner. These elements all target the core definition of reciprocity as a dynamic, well-balanced process of mutual, equal or complementary social and emotional interaction and sharing with another person (20, 21).

Previous studies with the IDT showed poorer reciprocal behavior in autistic children and adolescents compared to non-autistic peers (2, 3). Autistic Participants showed a strong preference for drawing their own objects and contributed less to examiner-initiated drawing objects. The IDT distinguished effectively between autistic and non-autistic youth (with average intellectual ability or with mild intellectual disabilities). Moreover, the test is also sensitive for gender differences both in autistic and neurotypical children (2, 3), which is highly relevant given the rates of camouflaging behavior in autistic females (22).

The sensitivity of the IDT to distinguish autistic and non-autistic youth can be attributed in part to the absence of explicit verbal guidance and the required spontaneity of the (non-verbal) interaction with the examiner. Autistic Individuals with average or high intelligence (from here on “average intelligence”) tend to rely more heavily on their verbal skills and scripted knowledge of social situations to solve social problems (12). Unlike the ADOS, the IDT does not provide clear verbal instructions nor a script to follow, thus enhancing the likelihood that social impairments of autistic participants will become visible (23–25).

In the current study we examined whether the IDT is also sensitive to differences in reciprocal behavior of autistic and non-autistic adults. We expected to replicate previous findings in autistic children, and confirm that also autistic adults show lower quality of reciprocal behavior than non-autistic adults, in particular with regard to the frequency of meaningful contributions to objects initially drawn by the examiner. Furthermore, we explored whether the IDT outcome correlated with the severity of autistic traits, estimated cognitive abilities (receptive verbal ability), age, and gender.

## Methods

### Participants

Participants of this study comprised 37 adults (age range 20–65 years): 19 autistic participants (13 males, 6 females) and 18 non-autistic participants (11 males, 7 females). All participants were born in the Netherlands and had the Dutch nationality (see Table 1). Initially 40 participants enrolled, but one non-autistic participant and two autistic participants were excluded because of incomplete data. Participants were recruited *via* VU University in Amsterdam and three health care institutions (in Amsterdam, Utrecht and Veldhoven). This study included only two participants with a co-occurring disorder, one participant with ADHD in each group. Independent psychiatrists and psychologists (who were not involved in the current study) established the diagnoses of the autistic participants according to DSM-IV-TR or 5 criteria (1) prior to recruitment. The diagnostic process included hetero anamneses as well as psychiatric and neuropsychological examinations.

**Table 1:** Descriptives for the ASD and TD groups.

	**ASD (*n* = 19)**		**TD (*n* = 18)**		**Total group (*N* = 37)**		**Group differences**
	***M (SD)***	**Range**	***M (SD)***	**Range**	***M (SD)***	**Range**	
Gender (n male; n female)	13; 6		11; 7		24; 13		*ns*
Age (in years)	34.0 (13.1)	20–65	32.6 (12.5)	20–62	33.3 (12.7)	20–65	*ns*
PPVT (Verbal receptive IQ)	109.4 (6.9)	100–129	104.1 (8.5)	89–117	106.8 (8.0)	89–129	ASD > TD
Educational level							*ns*
High	95%		89%		92%		
Middle	5%		11%		8%		
Low	0%		0%		0%		
Employment							*ns*
Paid	42%		61%		59%		
Student	58%		39%		41%		
AQ-28 (Autism Quotient)	78.1 (14.6)	53–107	45.3 (9.4)	32–65	62.1 (20.6)	32–107	ASD > TD
Additional mental health problems							*ns*
ADHD	1		1		2		
ADD	0		1		1		
Depression	1		0		1		
Dyslexia	1		3		4		

Receptive verbal ability was measured with the Peabody Picture Vocabulary Test-III-NL [PPVT-III-NL (26)]. The PPVT was chosen due the brief administration time. One non-autistic participant and two autistic participants could not participate in the PPVT, but had intelligence scores within the normal range on the Wechsler Intelligence Scale for adults WAIS-IV (27) (Verbal IQ, ranging 100–129) (see for descriptions of participants Table 1). Correlations between PPVT scores and verbal IQ scores based on the WAIS are high (28).

All participants were well-educated and were students or had a paid job. There were no differences between the groups in the number of university students (ASD 42.1%, no ASD 38.9%) or having a paid job (ASD 57.9%, no ASD 61.1%). As expected, autistic participants showed higher levels of autistic traits, indicated by a higher score on the Autism Quotient-28 [AQ-28 (29)], compared to the non-autistic adults [*F*_(1, 35)_ = 64.97, *p* = 0.000, $\eta_{p}^{2}$= 0.65] (see Table 2).

**Table 2:** AQ-28 scores of the ASD and TD group and differences.

	**ASD (*n* = 19)**		**TD (*n* = 18)**		**Group differences**
	**M (SD)**	**Range**	**M (SD)**	**Range**	
Social skills	20.1 (5.5)	12–28	10.7 (3.3)	7–19	*p* < 0.001
Routine	11.4 (2.8)	7–16	6.7 (2.0)	4–10	*p* < 0.001
Switching	12.2 (2.4)	8–16	7.1 (2.0)	4–12	*p* < 0.001
Imagination	20.8 (4.7)	11–29	12.8 (3.9)	8–22	*p* < 0.001
Numbers, patterns	13.6 (3.5)	6–20	7.9 (2.9)	5–16	*p* < 0.001
Social behavior	56.4 (10.3)	40–77	33.2 (6.9)	24–52	*p* < 0.001
AQ total score	78.1 (14.6)	53–107	45.3 (9.4)	32–65	*p* < 0.001

In the ASD group, four participants scored below the cut off on the AQ-28 (e.g., not in the clinical range), which can occur in autistic adults and average or above average intelligence (6). They were not excluded from the study because they all had received a clinical diagnosis of ASD.

A sample size of at least 22 is necessary for detecting a effect with a power level of 80% and a significance level of 0.05, using a sample size calculator G^*^Power, based on the previously found effect size of $\eta_{p}^{2}$ = 0.31 (2, 5).

### Procedure

After signing an informed consent form, participants were invited to come to the University to participate in the study. Participants recruited via the Health Care institutions were already tested with the PPVT and AQ as part of the diagnostic procedure. The PPVT and AQ were additionally administered to participants recruited through the university. The IDT was administered in all participants in a test room. The average duration was 10 min. Community members were not involved in the development of the IDT, but did help with the procedural refinement.

### Measures

#### Autism Quotient-28

The Dutch version of the AQ-28 [AQ-28, Hoekstra et al. (29)] was used to measure autistic traits. The AQ-28 is a 28-item self-report screening measure of ASD traits that is strongly correlated with the outcome of the original AQ with 50 items and has good sensitivity and specificity (28). The AQ-28 is a shortened version of the original AQ and consists of 28 questions in 6 subscales: 1. Social skills/abilities, 2. Routines, 3. Switching (attention), 4. Imagination, 5. Numbers and patterns (fascination), 6. Social behavior. For instance, one of the questions in the subscale “Imagination” is: “Reading a story, I find it difficult to work out the character's intentions” or in the subscale “Attention switching”: “I like to carefully plan any activities I participate in.” Answers to the questions are scored on a 4-point scale ranging from definitely agree to definitively disagree. The test provides a total score and 6 subscale scores. A total score at or above 65 means a score in the clinical range. Higher scores indicate higher levels of ASD traits.

#### The Peabody Picture Vocabulary Test

The PPVT (30) was used to test receptive vocabulary. The test consists of a series of pictures and is suitable for a wide age range (2–90 years). The participant has to match an orally given word to a picture. The reliability of the PPVT with split-split half and retest administration is excellent and the construct and content validity is good. The validity of the PPVT is evidenced by strong correlations between PPVT scores and overall intelligence (31).

#### The Interactive Drawing Test

The Interactive Drawing Test [IDT (5)] assesses real-life reciprocal behavior by coding whether and how often a participant draws objects on a shared piece of paper in mutual collaboration with the examiner. The examiner is instructed to elicit spontaneous natural reciprocal behavior and to facilitate reciprocal behavior, without directive verbal suggestions to the participant. The examiner has received specific instructions what to draw and when. The IDT was developed for an age range of 6–18 years. As the task is mostly non-verbal, no procedural adaptations for adults were necessary. At the start the examiner and the participants sit opposite each other, on either side of a blank rectangular paper (A-3 size). Both have different colored pens and the examiner's only instruction at the start of the test is: “We are going to draw together.” The examiner is instructed to draw in his first turns a simple picture of a house (floor, walls and roof) and to start with a basic horizontal line (floor) in his first turn, and then to push and rotate the paper back to the participant.

**Figure 1:**
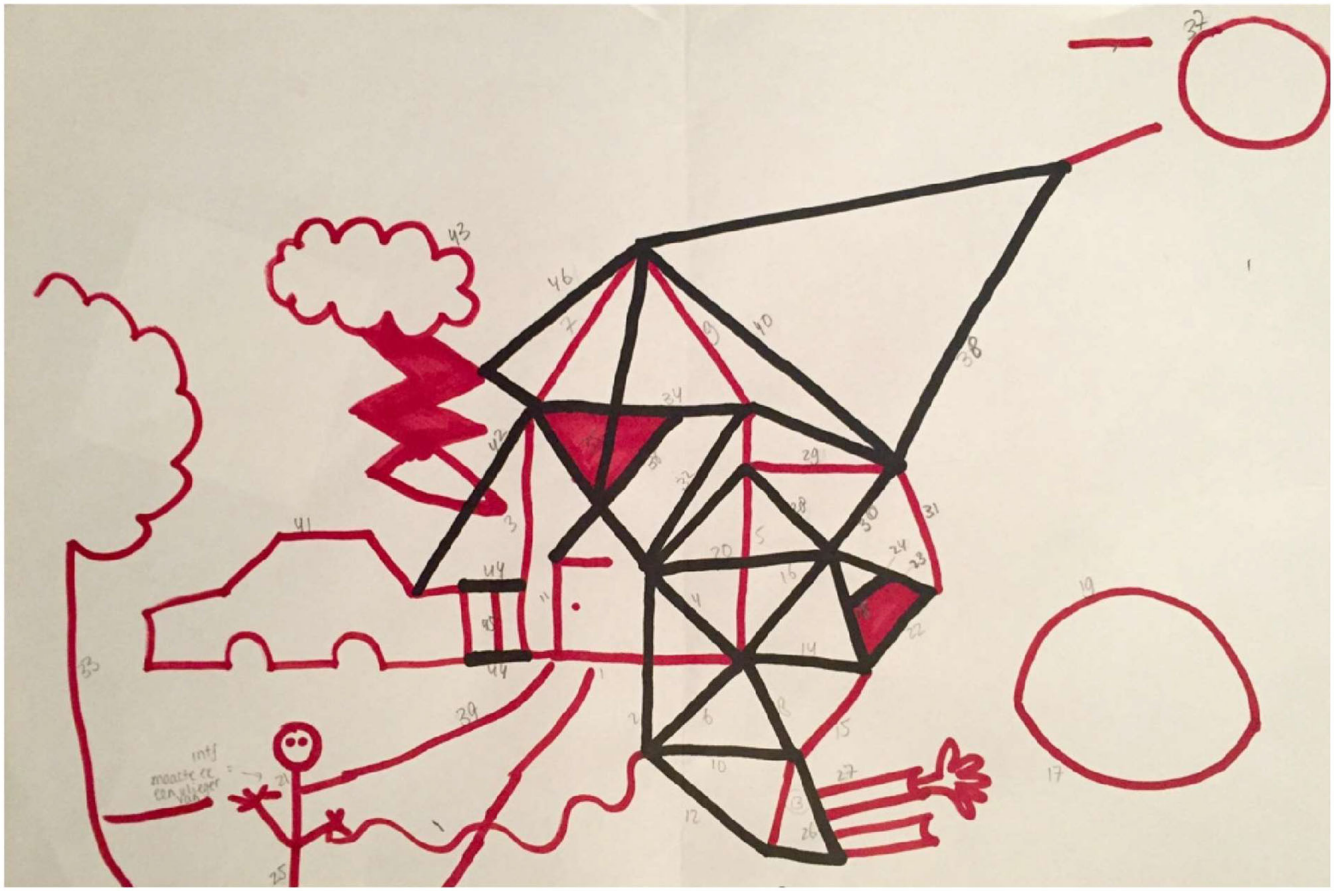
IDT drawing by an autistic woman (age 40 years, higher vocational education, paid job, total AQ score: 107) who changed the house of the examiner into a futuristic building and did not contribute to the examiner's objects.

**Figure 2:**
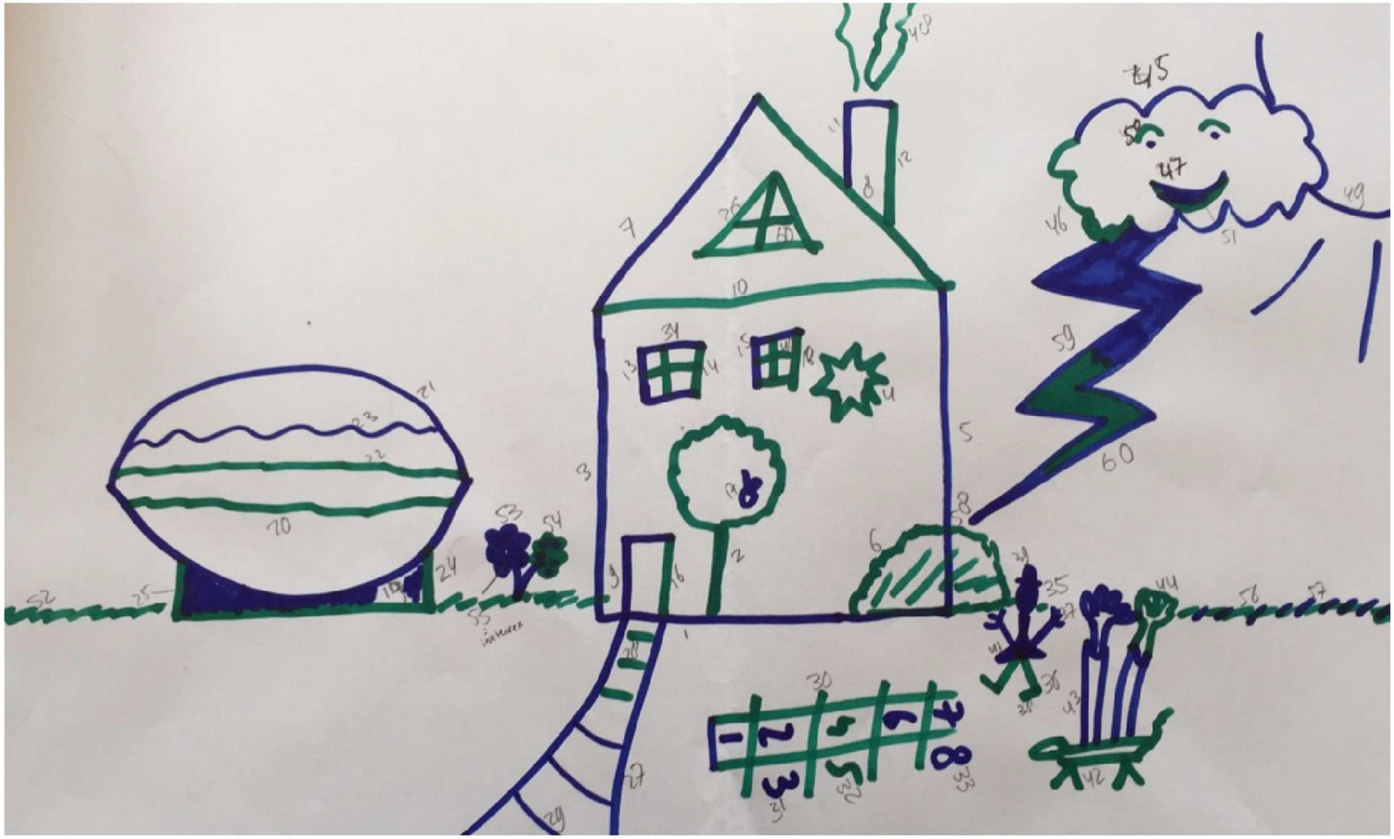
IDT drawing by a non-autistic man (age 26 years, master degree, paid job, total AQ score: 41), who regularly contributed to the objects of the examiner.

When a participant asks what to draw, the examiner answers: “You may draw anything you like.” According to the IDT protocol, the examiner continuously draws unfinished objects (e.g., drawing half a window in the house) in order to elicit reciprocal behavior and to facilitate contributing to each other's objects. Following the picture of the house, the examiner draws a bow next to the house. This bow can become anything, allowing the participant to decide what the bow will be (e.g., a pond, a motorcar, a ball, see Figure 1). The participants are free to draw anything they like, including idiosyncratic or eccentric own objects (e.g., drawing a geometric figure after the examiner draws the bow, see Figure 2), or adding appropriate elements to existing objects (e.g., drawing their own car on the highway initiated by the examiner), or contributing elements to the examiner's object (e.g., after the examiner has initiated a car, adding a steering wheel to the car).

Halfway through the procedure, the examiner interferes with the participant's drawings by adding three different elements. First, the examiner adds an *interfering* element that changes the nature of the participant's drawing but fits within context (e.g., turning a figure of a boy into a girl by adding a dress). Second, the examiner adds an *absurd* element (e.g., adding arms to an airplane), and third, the examiner adds a *damaging* element: a bolt of lightning hitting an object drawn by the participant (if the “object” is human or an animal, the bolt of lightning is drawn in the vicinity). The second element (two arms, one with and one without a hand) and the third element (bolt of lightning) are standard. After the participant's response to the final element, the examiner asks if the participant thinks the drawing is finished or whether it needs another addition. The participant is allowed to make this final addition. The IDT takes ~10 min in total. After finishing the IDT, the participant is asked to rate whether he/she liked taking part in the drawing task on a 5-point scale (with smileys) ranging from very much to not at all.

#### Scoring the IDT

With the IDT four elementary aspects of reciprocal behavior achieved at the age of 6 years are objectively assessed by counting their occurrence: 1. Turn-taking (pushing and rotating the paper back to the examiner, i.e., is the participant able to give and take turns adequately?), 2. Reciprocal interaction (is the participant able to contribute to a mutual goal?), 3. Reciprocal interaction in the other's initiative (contributing to objects drawn first by the examiner, i.e., is the participant able to understand the intention of others and adjust own actions accordingly?), and 4. Reciprocal flexibility (does the participant accept the three interfering drawing elements of the examiner, for instance by incorporating them into his own drawing?). Scales 3 and 4 are considered to reflect more advanced levels of reciprocity, including the flexibility to deal with interfering or complex additions to one's drawing. The scale “Reciprocal interaction in the other's initiative” was the most sensitive outcome in previous studies with children and adolescents with ASD (3, 4).

The number of turns is counted and used to calculate the proportion of scale scores of “Turn- taking,” “Reciprocal interaction,” and “Reciprocal interaction in the other's initiative” in relation to the total number of turns. The proportion of the scale score of “Reciprocal flexibility” (three inputs) is calculated by dividing the scale score by three. A total reciprocity score is then calculated by adding the four proportion scales, and this total score reflects the level of reciprocal behavior in general. A “Total score” below 2.50, “Turn-taking” score below 0.71 and a “Reciprocal interaction in other's initiative” score below 0.32, are performances in the clinical range according to the norms for children and adolescents (5). The IDT has good to excellent reliability, and good validity to distinguish between individuals with and without ASD in the age range of 6–18 years with a sensitivity of 0.82 and specificity of 0.23 for “Total score,” 0.74 and 0.34 for “Turn-taking” and 0.80 and 0.23 for “Reciprocal interaction in other's initiative (3).”

## Results

### Ratings of IDT Participation

Of the autistic adults 52.6% did not like participation of the IDT very much (i.e., a neutral or negative rating) in contrast to only 11.1% of non-autistic participants: a significant difference [*F*_(1, 35)_ = 8.56, *p* = 0.006, $\eta_{p}^{2}$= 0.20]. In the total group, we found a mild positive correlation of this rating with the “Total score” (*r* = 0.38, *p* < 0.05) and a stronger correlation with “Turn-taking” (*r* = 0.51, *p* < 0.01). No significant associations were found between the IDT rating and other IDT measures (*r*'s between 0.15 and 0.31). When analyzing correlations in the separate groups, we only found a strong correlation of the rating with “Turn-taking” (*r* = 0.58, *p* < 0.01) in the ASD group.

### Group Differences

Autistic and non-autistic participants did not differ in age [*F*_(1, 35)_ = 0.11, *p* = 0.75] or gender [*F*_(1, 35)_ = 0.21, *p* = 0.65]. Autistic participants had better receptive verbal ability [*F*_(1, 35)_ = 4.4, *p* = 0.04, $\eta_{p}^{2}$= 0.11] compared to non-autistic participants.

#### Reciprocal Behavior

Differences in reciprocal behavior in general and in different aspects of reciprocal behavior were tested using univariate Ancovas, controlling for verbal receptive IQ measured with the PPVT. As hypothesized, autistic participants had lower scores than non-autistic participants on the IDT “Total score” [*F*_(1, 30)_ = 5.28, *p* = 0.029, $\eta_{p}^{2}$= 0.15], and on the scale 'Reciprocal interaction in other's initiative' [*F*_(1, 30)_ = 11.77, *p* = 0.002, $\eta_{p}^{2}$= 0.28]. Figure 1 (autistic participant) and Figure 2 (non-autistic participant) illustrate the difference in reciprocal drawing, with the non-autistic participant joining the examiner in drawing the house. No significant group differences were found on the IDT scales: “Turn-taking” (*p* = 0.10), “Reciprocal interaction” (*p* = 0.35), and “Reciprocal flexibility” (*p* = 0.26). Of the three input scores that contribute to the scale “Reciprocal flexibility,” two scores, accepting the absurd (*p* = 0.88) and the destructive input (*p* = 0.57), were similar between groups. However, the interfering input (the researcher changes the nature of participant's object) was significantly less accepted by autistic adults [*F*_(1, 35)_ = 5.04, *p* < 0.05, $\eta_{p}^{2}$= 0.13].

### Correlations of Participant Characteristics With IDT Scores

#### Receptive Verbal Abilities and Age

In the total group, no significant correlations were found between the scales scores and “Total score” of the IDT and verbal IQ (*r*'s ranging from−0.003 to 0.05) or age (*r*'s ranging from−0.31 to 0.17; see Table 3). We also did not find significant correlations of verbal IQ with the Total IDT score when analyzing the groups separately (autistic participants *r* = −0.03, non-autistic participants *r* = 0.35).

**Table 3:** Correlations between IDT scores, AQ-28, PPVT and age in the total group.

	**ASD traits (AQ-28)**	**Receptive verbal ability (PPVT)**	**Age**
Turn-taking	−0.39*	−0.003	0.054
Reciprocal interaction	−0.39*	0.033	0.004
Reciprocal interaction in other's initiative	−0.49**	0.038	−0.10
Reciprocal flexibility	−0.26	0.049	−0.31
IDT “Total score”	−0.49**	0.037	−0.18

**p <0.01; *p <0.05.

#### Autistic Traits

Negative correlations were found between the AQ total score and total IDT score (-0.49, *p* < 0.01) and all reciprocity scales, except “Reciprocal flexibility” (see Table 3) in the total group of participants, indicating that more autistic traits corresponded with lower levels of reciprocal behavior and especially with less reciprocal interactions in other's initiative (-0.49, *p* < 0.01).

In the ASD group, a lower AQ scale score on switching skills (e.g., “I have difficulty with switching attention or switching between activities”) correlated negatively with “Reciprocal interaction” (-0.52, *p* < 0.05). A lower AQ scale score on imagination (e.g., “Reading a story I find it difficult to work out characters' intentions”) corresponded with higher scores on the IDT “Total score” (-0.53, *p* < 0.05) and “Turn-taking” (-0.52, *p* < 0.05) (Table 4). In the no ASD group, no significant correlations were found between the AQ scale scores and the IDT scores (*r's* ranging from −0.03 to −0.45), suggesting that the IDT is not sensitive to autism-relevant differences in neurotypical samples.

**Table 4:** Correlations between IDT scale scores and AQ scale scores in the ASD group.

	**IDT Total score**	**Turn-taking**	**Reciprocal interaction**	**Reciprocal interaction in other's initiative**	**Reciprocal flexibility**
AQ total score	−0.38	−0.33	−0.36	−0.08	−0.21
Social skills	−0.42	−0.37	0.23	0.14	−0.31
Routine	−0.14	−0.05	−0.32	−0.16	−0.08
Switching	−0.08	−0.01	−0.52*	−0.08	−0.16
Imagination	−0.53*	−0.52*	−0.41	−0.37	−0.21
Numbers, patterns	−0.04	−0.07	0.00	0.31	−0.14
Social behavior	−0.41	−0.35	−0.45	−0.17	−0.17

*p <0.05.

## Discussion

The present study aimed to test the sensitivity of the Interactive Drawing Test to differences in reciprocity in autistic and non-autistic adults. Autistic adults showed less overall reciprocal behavior (lower “Total score”) and in particular contributed less frequently to the examiner's drawing objects compared to non-autistic adults (Table 5). Autistic adults were thus less able to ensure an equal balance between drawing together in their own objects and in the objects drawn by the examiner, and consequently showed a lower quality of reciprocity. These findings are in line with the outcome of the IDT studies in autistic children and adolescents (3, 4). Similarities in the ability of autistic and non-autistic adults were found in the ability to reciprocate in general (i.e., the quantity of reciprocal interactions irrespective of who initiated the drawing interaction) (Table 5), and in “Turn-taking” and “Reciprocal flexibility.” These findings were in contrast with the outcome of the previous child and adolescent IDT studies and indicate that autistic adults might outperform autistic youngsters on more basic aspects of reciprocal behavior. Alternatively, the IDT might not be sensitive enough to pick more fine-grained reciprocity differences in autistic adults, like many social cognitive and behavioral measures.

**Table 5:** IDT scores of the ASD and TD group, controlled for verbal receptive IQ.

	**ASD (*n* = 19)**		**TD (*n* = 18)**		**Group differences**
	**M (SD)**	**Range**	**M (SD)**	**Range**	
Turn-taking	0.87 (0.08)	0.11–1.00	0.94 (0.08)	0.68–1.00	*ns*
Reciprocal interaction	0.76 (0.14)	0.00–1.00	0.82 (0.16)	0.40–1.00	*ns*
Reciprocal interaction in other's initiative	0.27 (0.13)	0.00–0.53	0.42 (0.16)	0.05–0.62	*p* = 0.002 *$\eta_{p}^{2}$* = 0.28
Reciprocal flexibility	0.49 (0.35)	0.00–1.00	0.65 (0.37)	0.00–1.00	*ns*
Total score	2.45 (0.42)	0.61–2.98	2.82 (0.65)	1.13–3.47	*p* < 0.05 *$\eta_{p}^{2}$* =0.15

The lower scores on the IDT scale “Reciprocal interaction in other's initiative” found in autistic youth *and* adults could be partly explained by a shared limited understanding of others' intentions observed in autistic individuals (24, 32). A limited understanding of the examiner's intentions might have caused the adults with ASD to refrain from contributing to the examiner's objects and to draw their own instead. The negative correlation between self-reported problems with understanding intentions of others (on the AQ) and overall reciprocal behavior in autistic participants underlines that participants were aware of their shortcomings, and this awareness was reflected in their real life behavior.

Autistic characteristics such as the difficulty to deal with unexpected situations and the preference for predictability and having control (33) might also have stimulated participants with ASD to draw their own objects, or to change the object of the examiner into his/her own object (see Figure 1). As expected, more autistic traits, reported by participants on the AQ-28 (29), correlated with lower reciprocity scores on all subscales except for Reciprocal flexibility. This outcome matches previous IDT studies in youth (3, 4), where the severity of autistic traits, reported by caretakers on the Social Responsiveness Scale [SRS (34)], correlated negatively with the level of reciprocal behavior. These findings in adults and in children suggest acceptable convergent validity of the IDT, considering that the AQ and the SRS (while they assess a broader construct than the IDT) both measure social interaction skills. Further research with a larger sample of adults is necessary to confirm the convergent validity of the IDT.

Age and verbal intelligence (measured with the PPVT) were not associated with reciprocal behavior within the adult groups (Table 3), confirming our previous findings in the child and adolescent samples (3, 4). Furthermore, even though autistic adults had a higher mean receptive verbal IQ than non-autistic participants, they did not perform better on the IDT even when controlling for verbal intelligence. This outcome underlines the efficacy of the non-verbal IDT properties which do not allow compensation or masking of social impairments by strong verbal cognitive abilities (15). While the social rule to engage with other people may be relatively simple to learn for autistic adults, the unusual interaction of making a joint drawing will not likely facilitate this kind of compensation.

Motivation for learning social skills and improving social functioning might explain the better performances on more basic aspects of reciprocal behavior of autistic adults compared to autistic youth. Studies have found that autistic adults become more motivated for social interactions (35). Motivation seems to improve social skills in early adulthood, for instance during their study or in jobs (36, 37). Autistic adults probably are also more inclined to imitate others (38) and may therefore show, for instance, more adequate turn-taking behavior; however, it should be noted that imitation problems are not universal in autism (39). Autistic participants accepted various types of input to their own objects (“Reciprocal Flexibility”) with obvious visual clues for a suitable response (e.g., an unfinished bolt of lightning that could be completed). However, they were not as accepting (i.e., not as flexible) as non-autistic participants if the input changed the nature of their object (e.g., changing their motorcar into a fire engine). An explanation could be that some autistic adults had a fixed idea about the object they had drawn and were more reluctant to divert from their original plan. An alternative interpretation might be that individuals with autism struggled to understand the intent of the observer in terms of the changes they sought to make, and what they were transforming their object into. However, evidence against this alternative lies in the fact that the ASD group actually had higher PPVT scores.

Autistic adults did not appreciate the IDT as much as non-autistic adults. Some autistic adults seemed to find the conditions of the IDT confusing and unclear, this might have diminished their motivation for participating. This may be related to the “double empathy problem,” referring to the breach in natural reciprocity between people of different dispositions, like those with and without autism (40). For instance, two autistic participants told the examiner half way the administration that they did not want to continue because they did not understand what was expected of them, and this became too frustrating and stressful. Their failure to complete the test is an example of how and why an autistic individual might fail to show adequate reciprocity in real-life situations. Because of incomplete data they were excluded from the study. The evaluation of the test by autistic adults only correlated with “Turn-taking,” a basic aspect of reciprocity. This finding suggests that the quality of their reciprocal behavior (i.e., contributing to other's initiative) was not influenced by their level of appreciation or the motivation to participate in the IDT.

It is striking that the IDT, which requires non-verbal reciprocal behavior at the level of a 6-year-old child, is able to show subtle differences between normally intelligent autistic and non-autistic adults. The IDT's sensitivity for reciprocity impairments even in normally intelligent adults seems due to its unstructured, non-rule governed, unfamiliar conditions, its non-verbal method, and the focus on reciprocal interactions in the other's initiative. In contrast to more structured observational tasks like the ADOS, it is likely much more difficult for autistic adults to work out what the experimenter is trying to measure in their behavior. Importantly, this difficulty in understanding is a difficulty in understanding the intent of the experimenter, both in drawing and in terms of what he/she is seeking to measure, not simply a difficulty understanding the basic instructions, for we excluded participants that became frustrated as they did not grasp the task and disparities weren't across the boards, but rather in the specific facets of reciprocity.

Furthermore, the IDT requires quick and spontaneous responding that does not allow premeditated decision making or pauses to reflect on intention, necessitating quick reaction and fluent collaboration. Intuitive understanding of the intentions of others is needed if participants choose to contribute to the examiner's object. Spontaneous responding seems particularly challenging for autistic individuals (25, 41–43). In case autistic individuals and average intellectual ability are given sufficient time for conscious decision making or metalizing (32, 44, 45). or they can rely on their verbal abilities (12, 46), they may perfectly well-understand and react adequately on others' intentions and emotions. In addition the current access to real-life social reciprocal engagement could also inform on the social abilities of a range of other psychiatric problems, including ADHD, depression, anxiety and personality disorders (12). The low specificity of the IDT needs to be studied in detail, and should currently be considered a limitation of the IDT.

The present study has several limitations. First, as the study only comprised 13 female adults (6 autistic and 7 non-autistic), gender differences in reciprocal behavior could not be analyzed. This is unfortunate because various studies, including our own IDT study with autistic youths, indicate that autistic females seem more motivated and often more socially engaged with others than autistic males (47–50) though gender differences are not consistent (51). Further research with more females in a larger sample is necessary for investigating gender differences in adults. Second, our findings cannot be generalized because our sample was small, females were underrepresented, and participants were normally intelligent and without co-occurring disorders. Future studies will need to replicate our findings in larger samples, including more restricted age ranges. Also, co-occurring disorders often occur in autistic adults and could influence their social functioning (52, 53). Third, the reliability of the IDT also depends on the expertise of the examiner. Despite good training and a clear protocol, the interaction process during administration will inevitably contain subjective elements. The fact that we had only one (female) examiner could also affect results. In future IDT studies the influence of a mixed or same gender dyad needs to be investigated. Finally, making a drawing may not be an appropriate activity for adults. While the unusual nature of the activity has many benefits, it may be relevant to study more reciprocity in more age-appropriate activities. However, the fact that the test is fluent and does not allow for verbal compensation may override these reservations.

This study reveals subtle reciprocal impairments in autistic adults and average intelligence, and suggests that the IDT could contribute to the diagnostic process of ASD in adults. It should be noted though that some normally intelligent autistic individuals will still pass the level of reciprocity assessed with the IDT. Therefore, in the diagnostic process, the IDT should be used in combination with multiple measures (interviews and questionnaires) to get an overall picture of functioning. Crucially, IDT studies with larger and randomly matched samples, including individuals with co-occurring disorders and an intellectual disability and male and female examiners, are needed to confirm and elaborate our findings. Future studies may also add motion tracking sensors to the IDT, which may be able to establish observer-independent measures of reciprocity (54).

## Data Availability Statement

The raw data supporting the conclusions of this article will be made available by the authors, without undue reservation.

## Ethics Statement

Ethical review and approval was not required for the study on human participants in accordance with the local legislation and institutional requirements. The patients/participants provided their written informed consent to participate in this study.

## Author Contributions

TB, HK, and SB developed the measure and the design and writing of the manuscript. MV and ASp coordinated the recruitment and assessment. ASc and RJ wrote sections in final draft of the manuscript. All authors contributed to manuscript revision, read, and approved the submitted version.

## Conflict of Interest

SB and TB receive royalties from the hogrefe publication of the child version of the Interactive Drawing Test. The remaining authors declare that the research was conducted in the absence of any commercial or financial relationships that could be construed as a potential conflict of interest.

## Publisher's Note

All claims expressed in this article are solely those of the authors and do not necessarily represent those of their affiliated organizations, or those of the publisher, the editors and the reviewers. Any product that may be evaluated in this article, or claim that may be made by its manufacturer, is not guaranteed or endorsed by the publisher.
